# Factors influencing college students’ willingness to participate in community volunteer services for the older adults in China

**DOI:** 10.3389/fpubh.2026.1763821

**Published:** 2026-07-29

**Authors:** Jing Shen, Xiaohu Duan, Shengyu Guo

**Affiliations:** School of Economics and Management, Changsha University, Changsha, China

**Keywords:** capacity building, college students, community volunteer services for the older adults, influencing factors, participation willingness

## Abstract

**Objective:**

To investigate college students’ willingness to participate in community volunteer services for the older adults and identify its associated influencing factors.

**Methods:**

A questionnaire survey was conducted from May to October 2025 through multiple channels, including online platforms, telephone interviews, and offline paper distribution. A total of 1,372 valid questionnaires were collected and analyzed.

**Results:**

In total, 38.27% of participants had previously engaged in community volunteer activities for the older adults, while 77.77% expressed willingness to participate in such initiatives. Significant associations (*p* < 0.05) were detected between students’ participation willingness and the following variables: gender, household registration type, capacity building for voluntary older adults care services, university support, social support, altruistic spirit, awareness of respecting and caring for the older adults, and communication skills. Multiple logistic regression analysis indicated that several factors positively predicted college students’ willingness to participate in community volunteer services for the older adults, including female gender (OR = 1.443), strong service capacity building (OR = 12.526), and sound university institutional support (OR = 2.063).

**Conclusion:**

Although community volunteer services for the older adults are widely recognized and accepted among college students, their overall participation willingness remains insufficient. Universities and relevant institutions should establish long-term operational mechanisms, optimize service capacity-building systems, and strengthen institutional support, and these measures can effectively enhance college students’ enthusiasm for participating in community-based older adults care volunteer services.

## 1 Introduction

Global population aging criteria stipulate that a country enters an aging society when the population aged 60 and above accounts for 10% of the total population, or the population aged 65 and above accounts for 7%. China exceeded the 10% threshold for the population aged 60 and above as early as 2000, marking its official entry into an aging society. According to statistics released at the end of 2024, China’s population aged 60 and above reached 310.31 million, accounting for 22.0% of the total population; those aged 65 and above numbered 220.23 million, accounting for 15.6%. The medium-variant projection of the World Population Prospects 2024 issued by the United Nations predicts that by 2050, China’s population aged 60 and above will exceed 500 million, accounting for approximately 40% of the total population.

China is among the countries experiencing the most rapid population aging worldwide. The scale of its older adults population is enormous, and social problems derived from this demographic trend will become increasingly prominent in this century. These challenges include high old-age dependency ratios, older adults crime, senior suicide, older adults care shortages, and pension fund pressures. Among these issues, older adults care has become an urgent and critical bottleneck restricting China’s long-term social and economic development.

Against the backdrop of China’s current pension development, the most prominent challenge lies in the phenomenon of “aging before affluence”: China has yet to attain the economic development level of developed economies, which restricts the country from providing universal formal older adults care services for all senior citizens. The construction of China’s older adults care service system is currently confronted with multiple constraints, including insufficient service supply, a critical shortage of professional practitioners, inadequate policy backing, and the declining functional capacity of family-based older adults care. Conjointly, these bottlenecks render the existing service system incapable of fully catering to the diversified demand for social older adults care among the older adults population. In practice, the national pension system struggles to meet the demands of the rapidly expanding older adults population. The high dependency ratio further imposes a heavy financial burden on the younger generation. Inadequate older adults care services also reduce the quality of life for many seniors. Alarmingly, some rural older adults individuals have even resorted to suicide due to insufficient pension security and medical resources. To address these pressing challenges, scholars and policymakers have been exploring optimal pension models to alleviate China’s growing older adults care crisis.

At present, China’s older adults care system can be categorized into three primary models: family-based care, community-based care, and institutional care. Each model aims to balance resource allocation, cultural traditions, and socioeconomic realities to achieve sustainable older adults care (1). According to data from the National Bureau of Statistics, by the end of 2023, China had 8.201 million institutional older adults care beds, covering 2.76% of the older adults population. This proportion aligns closely with the national “9,073” pension policy framework, which proposes that 90% of the older adults rely on family care, 7% on community services, and 3% on institutional care. Within this framework, community-based home care has become a core pillar and the dominant model in China’s older adults care system (2).

A community is defined by three interrelated elements: a specific geographical area, a resident population, and a shared sense of identity. As the basic unit of social governance, the community functions as a microcosm of society. In community older adults care, the close linkage between communities and older adults families has formed two mainstream operational models: the “community–family integrated pension model”, in which families remain the core of care delivery within the community; and the “mutual-aid older adults care model”, in which older adults residents provide support to one another within the community.

This dual model offers distinct advantages. Firstly, as every household is embedded within a community, improving community older adults care systems can delay the demand for institutional older adults care. Secondly, enabling older adults to age in place allows them to stay in familiar living environments and improves their overall care experience. Thirdly, this model compensates for the limitations of traditional family care by sharing care responsibilities and alleviating the burden on younger generations. Despite remarkable progress, China’s community older adults care system still faces prominent challenges, particularly the large older adults population base and the severe shortage of professional social workers. Owing to factors including inadequate professional literacy and competence, poor occupational attractiveness, low public recognition, insufficient relevant policy support and limited volunteer participation, there remains a substantial shortage of the workforce engaged in social older adults care services across China at present. Recruiting, training, and retaining qualified service personnel has become an urgent priority.

Volunteer services serve as a vital approach to alleviating the talent shortage in modern older adults care. Community older adults care volunteer services refer to charitable activities in which volunteers provide free services to older adults residents relying on community platforms. Such services cover daily life care, health care, and psychological support, forming an indispensable component of the socialized older adults care service system.

College student volunteers constitute the backbone of China’s youth volunteer workforce, featuring strong ideological awareness and a high willingness to engage in social welfare initiatives (3). To clarify their role in community older adults care, this study conducted a random questionnaire survey of over 2,000 college students from May to October 2025. The research aimed to analyze their current participation status in community older adults care volunteer services, propose targeted optimization strategies, and provide empirical evidence and policy implications for promoting college student participation in community older adults care.

## 2 Theoretical background

This study is grounded in the Theory of Planned Behavior (TPB), a classic social psychological theory that explains individuals’ behavioral decision-making processes. Its theoretical origin derives from the Theory of Reasoned Action (TRA) proposed by Ajzen and Fishbein in 1973, which evolved from Fishbein’s Expectancy-Value Model of Attitude (4, 5). The TPB has been widely applied in multiple research fields, including tax compliance, pro-environmental behavior, volunteer participation, and electoral voting. Numerous empirical studies have confirmed that TPB significantly improves the explanatory power and predictive validity of behavioral intention and actual behavior.

**Figure 1:**
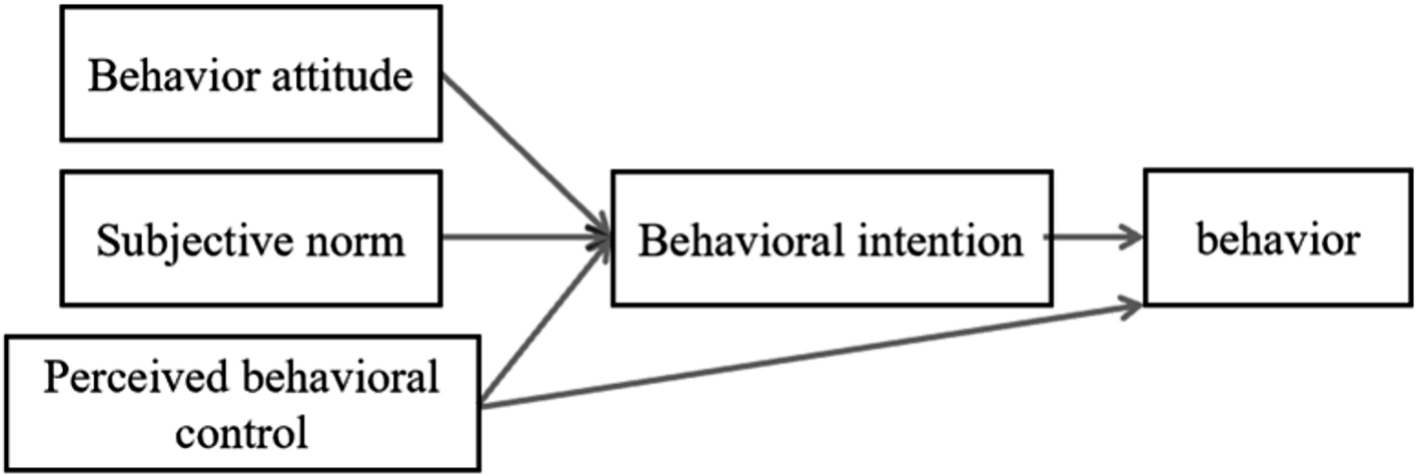
Schematic diagram of planned behavior theory.

The TPB comprises five core components: behavioral attitude, subjective norm, perceived behavioral control, behavioral intention, and actual behavior (Figure 1). According to TPB, individual behavior is not merely determined by behavioral intention; it is also constrained by personal capabilities, available resources, and external opportunities. Behavioral attitude refers to an individual’s positive or negative evaluation of performing a specific behavior. Subjective norm reflects individuals’ perceptions of whether significant others expect them to engage in a certain behavior, including perceived social pressure and the degree of compliance with important referents. Perceived behavioral control denotes individuals’ subjective judgment of their ability to perform a given behavior. Behavioral intention represents individuals’ direct tendency to conduct a specific behavior, while actual behavior refers to concrete behavioral performance. Notably, perceived behavioral control can directly influence actual behavior without the mediation of behavioral intention.

Although TPB provides a solid theoretical foundation for this study, it still has limitations in fully explaining college students’ willingness to participate in community older adults care volunteer services. For instance, TPB ignores objective constraints such as academic workload, transportation costs, and geographical distance, all of which substantially affect students’ volunteer participation intentions. Furthermore, existing literature indicates that past behavioral experience is one of the strongest predictors of future behavior, whereas the original TPB does not incorporate this variable. Existing studies have demonstrated that college students’ willingness to participate in volunteer services is comprehensively affected by a combination of multiple factors, including time constraints, individual characteristics, social support, incentive mechanisms, participation channels, service quality, and perceived social contribution (6, 7).

To address these deficiencies, this study expands the classical TPB framework by integrating relevant variables from previous studies. The extended influencing factors include college students’ individual characteristics, older adults care volunteer service capacity building, environmental support, and older adults care volunteer knowledge and skills. Based on this extended framework, the following research hypotheses are proposed:

*H1*: University students' personal characteristics, including gender, academic discipline, and place of origin, positively influence their willingness to participate in community-based older adults care volunteer service.

*H2*: Capability development in older adults care volunteering positively influences university students' willingness to participate in community-based older adults care volunteer service.

*H3*: Environmental support positively influences university students' willingness to participate in community-based older adults care volunteer service.

*H4*: Knowledge and skills related to older adults care volunteering positively influence university students' willingness to participate in community-based older adults care volunteer service.

## 3 Objects and methods

### 3.1 Objects

From May to October 2025, the research team collected data from Chinese university undergraduates via online, telephone, and in-person channels, strictly adhering to the principles of confidentiality and voluntary participation, covering 29 provinces, autonomous regions, and municipalities directly under the Central Government of China. Specifically, 806 questionnaires were distributed via the “Questionnaire Star” platform using snowball sampling, with 735 recovered; 811 individuals were interviewed by telephone (also snowball sampling through personal contacts) with 752 valid records; for in-person surveys, referring to Qiu and Wen (8), 31 provincial-level regions were divided into eastern, central, and western regions. According to the regional classification conventionally adopted in China’s official statistical reports, the eastern region comprises 11 provinces: Beijing, Tianjin, Hebei, Liaoning, Shanghai, Jiangsu, Zhejiang, Fujian, Shandong, Guangdong and Hainan. The central region includes 8 provinces: Shanxi, Jilin, Heilongjiang, Anhui, Jiangxi, Henan, Hubei and Hunan. The western region covers 12 provinces: Sichuan, Guizhou, Yunnan, Xizang, Shaanxi, Gansu, Qinghai, Ningxia, Xinjiang, Chongqing, Inner Mongolia and Guangxi. Among them, universities in the western region account for 27% of the total number of universities in China, those in the central region account for 24%, and those in the eastern region account for 49%. The team selected 12, 8, and 8 universities in the eastern, central, and western regions, respectively, distributing 197, 97, and 109 questionnaires and recovering 285. After excluding invalid questionnaires (due to logical contradictions or high answer repetition), 1,372 valid questionnaires were finally obtained, with an effective response rate of 68.1%. The regional distribution of valid questionnaires (30.3% eastern, 52.6% central, 17.1% western) was consistent with the actual distribution of Chinese universities, and the gender ratio (63.36% female) was consistent with the 63% female undergraduate enrollment rate released by the Ministry of Education of the People’s Republic of China in 2022.

This study was approved by the Ethics Review Committee of Changsha University (Approval No.: [006–2024-10], Date: October 20, 2024). All respondents were informed of the research purpose, procedures, anonymity, and right to withdraw. Written informed consent was obtained prior to questionnaire completion; for online surveys, voluntary submission was regarded as implied consent.

### 3.2 Methods

The research group utilized a “questionnaire titled College Students Participation in Community-Based Voluntary Service for the Older adults” to conduct their survey. In this study, university students refer to full-time undergraduate students who are currently enrolled in higher education institutions.

The survey consisted of four primary components: (1) Respondent Demographics: This section gathered basic information about participants, including gender, grade, major, origin type, average monthly household income, parental educational attainment, and other relevant details. (2) Capacity Building for Voluntary Older adults Care Services: This study investigated college students’ willingness and participation regarding publicity, training, and drills related to voluntary older adults care services over the past year. A score of 1 was assigned to respondents who had participated or were willing to participate, and a score of 0 to those who had not participated or were unwilling to participate. Scores from the two items were summed. A total score greater than 0 was recoded as 1, while a total score of 0 was recoded as 0. (3) Environmental support: it comprises two components: school-based support and social support. School-based support assesses the publicity, organizational efforts, and rewards provided by universities to promote community volunteer activities for the older adults. Scores are calculated using a binary system: “Yes” responses earn 1 point, while “No” or “Unclear” responses receive 0 points. A final score greater than 0 is standardized to 1, whereas a score of 0 remains unchanged. Social support evaluates societal awareness, acknowledgment, and attention toward volunteer activities conducted by college students. Similarly, scores follow a binary scoring system: “Yes” earns 1 point, and “No” or “Unclear” receives 0. Scores above 0 are converted to 1, while a score of 0 retains its value. (4) Knowledge and skills of voluntary service for the older adults: The research team employed a Knowledge and Skills Scale (using a Likert 5-point scoring system) to assess participants’ engagement in voluntary service for the older adults. This scale encompassed multiple dimensions, including the spirit of altruism, awareness of respect and care for seniors, communication abilities, daily caregiving techniques, psychological nursing skills and preventive health knowledge related to common geriatric conditions. The results demonstrated satisfactory internal consistency (Cronbach’s alpha = 0.796). To facilitate logistic regression analysis, the scores of all individual items were summed, and the results were dichotomized using the median as the cut-off point. Scores greater than or equal to the median were classified as “adequate,” while scores below the median were classified as “inadequate.” The “adequate” category was coded as 1, and the “inadequate” category was coded as 0.

### 3.3 Statistical analysis

The statistical significance level was set at *α* = 0.05, and *p* < 0.05 was regarded as statistically significant. Given the sample size of 1,372 valid questionnaires (*n* > 1,000), the sample mean was assumed to follow a normal distribution. SPSS 23.0 was used for chi-square (*χ*^2^) test and binary logistic regression analysis. Variables with *p* < 0.05 in the univariate chi-square test were included in the multivariate regression model.

This study adopted a two-stage analytical strategy. First, univariate logistic regression was performed on all potential influencing factors proposed in the research hypotheses. Second, multivariate logistic regression was conducted incorporating only factors with statistical significance (*p* < 0.05) in the univariate analysis.

## 4 Results

### 4.1 Basic information

Among the respondents, 883 were female (64.36%) and 489 male (35.64%). Of these, 204 were medical students (14.87%), while 1,168 were non-medical students (85.13%). Additionally, 805 respondents (58.67%) were in lower grades (freshmen and sophomores), and 567 (41.33%) were in higher grades (juniors and above). Regarding geographical background, 456 participants (33.24%) resided in city areas, whereas 916 (66.76%) hailed from rural or suburban regions. Family income distribution showed that 308 individuals (22.45%) had a monthly household income below 3,000 yuan, 635 (46.28%) reported 3,000–6,000 yuan, and 429 (31.27%) earned over 6,000 yuan. Finally, parental educational attainment revealed that 205 respondents (14.94%) had parents with primary school education or below, 520 (37.9%) had parents who completed junior high school, 399 (29.08%) had parents with senior high school (including technical secondary school) qualifications, and 248 (18.08%) had parents educated at the junior college level or higher (Table 1).

**Table 1:** Basic information table.

Characteristics	Grouping	Number of people	Constituent ratio
Gender	Female	883	64.36%
	Male	489	35.64%
Major	Medical specialty	204	14.87%
	Non-medical specialty	1,168	85.13%
Grade	Lower grade	805	57.67%
	Higher grade	567	41.33%
Origin type	City areas	456	33.24%
	Rural or suburban areas	916	66.76%
Average monthly household income (yuan)	<3,000	308	22.45%
	3,000–6,000	635	46.28%
	>6,000	429	31.27%
Parents’ education level (answer with the highest)	Primary school and below	205	14.94%
	Junior high school	520	37.9%
	Senior high school (including technical secondary school)	399	29.08%
	Junior college and above	248	18.08%

### 4.2 The current participation of college students in community volunteer services for the older adults

Of the 1,372 college students interviewed, 525 (38.27%) had participated in community volunteer service for the older adults, while 847 (61.73%) had not. Additionally, 1,076 (77.77%) expressed willingness to engage in such activities, whereas 305 (22.23%) indicated no interest in participating.

### 4.3 Univariate analysis of college students’ willingness to participate in community volunteer service for the older adults

Significant differences were observed in gender, origin type, capacity building for voluntary older adults care services, school support, social support, the spirit of altruism, awareness of respect and affection for the older adults, communication skills, and college students’ willingness to participate in community-based older adults volunteer services (*p* < 0.05). Conversely, no significant differences were found in major, grade, household monthly income, parental educational background, daily caregiving techniques, psychological nursing skills or preventive health knowledge related to common geriatric conditions in relation to this willingness (*p* > 0.05) (Table 2).

**Table 2:** Univariate analysis of college students’ willingness to participate in community volunteer service for the older adults.

Factor	Number of respondents	College students willing to participate in community volunteer service for the older adults		*χ* ^2^	*P*
		Number	%		
Gender				14.396	0.000
Male	489	352	66.5		
Female	883	715	81.0		
Origin type				4.581	0.032
City areas	456	370	81.1		
Rural or suburban areas	916	697	76.1		
Major				0.707	0.401
Medical specialty	204	154	75.5		
Non-medical specialty	1,168	913	78.2		
Grade				1.388	0.239
Lower grade	805	635	78.9		
Higher grade	567	432	76.2		
Average monthly household income (yuan)				1.536	0.215
Low-income	308	231	75.0		
Middle-income	635	497	78.2		
Higher-income and above	429	339	79.0		
Parental education level				0.712	0.399
Primary school and below	205	154	75.1		
Junior high school	520	404	77.7		
Senior high school (including technical secondary school)	399	315	78.9		
Junior college and above	248	194	78.2		
Capacity building for voluntary older adults care services				356.287	0.000
Good	1,091	926	84.9		
Lacking	353	141	39.9		
School-based support				72.448	0.000
Good	852	727	85.3		
Lacking	520	340	65.4		
Social support				27.649	0.000
Good	1,208	968	80.1		
Lacking	164	99	60.4		
The spirit of altruism				12.770	0.000
Good	566	467	82.5		
Lacking	806	600	74.4		
Awareness of respect and care for seniors				14.945	0.000
Good	505	421	83.4		
Lacking	867	646	74.5		
Communication abilities				9.701	0.002
Good	949	666	70.2		
Lacking	423	401	94.8		
Daily caregiving techniques				1.157	0.282
Good	705	540	76.6		
Lacking	667	527	79.0		
Psychological Nursing Skills				2.146	0.143
Good	678	516	76.1		
Lacking	694	551	79.4		
Preventive health knowledge related to common geriatric conditions				0.359	0.549
Good	663	511	77.1		
Lacking	709	556	78.4		

### 4.4 Multivariate regression analysis of college students’ willingness to participate in community volunteer service for the older adults

Taking willingness to participate in community volunteer service for the older adults as the dependent variable (1 = willing, 0 = unwilling), binary Logistic regression analysis was first performed with demographic information as control variables to obtain Model 1 (9). Then, based on univariate analysis results, the remaining six factors: capacity building for voluntary older adults care services, school-based support, social support, the spirit of altruism, awareness of respect and care for seniors, communication abilities were included as independent variables in the binary Logistic regression model to generate Model 2. The results showed that capacity building level had a significant positive predictive effect on college students’ willingness to participate in community older adults care volunteer services (OR = 12.526, *p* < 0.001, 95%CI:9.153–17.141). Although the OR value of this variable was relatively large, cross-tabulation tests revealed that the frequency in each cell was greater than 0, with no empty cells or extreme frequency distributions, indicating no complete or quasi-complete separation issues and robust model estimation. The high OR value was mainly attributed to the considerably higher proportion of willingness to participate among the group with high-capacity building (90.88%) compared with the low-capacity group (39.94%), with a significant difference between the two groups, resulting in a strong effect size. Meanwhile, school support also exerted a significant positive influence on participation willingness (OR = 2.063, *p* < 0.001, 95%CI: 1.490–2.856). In addition, the data indicated that female students exhibited higher willingness to participate in community older adults care volunteer services than male students (OR = 1.443, *p* = 0.024, 95%CI: 1.050–1.984). The effects of the remaining variables on willingness to participate in community older adults care volunteer services were not statistically significant (Tables 3, 4).

**Table 3:** Model 1: effects of demographic characteristics on college students’ willingness to participate in community volunteer service for the older adults (*n* = 1,372, −2LL = 1432.242, Nagelkerke *R*^2^ = 0.024).

Variable	VIF	B	P	OR	95%CI
Gender (female = 1)	1.010	0.494	0.000	1.639	1.262–2.128
Origin type (city areas = 1)	1.176	0.316	0.042	1.372	1.012–1.859
Major (medical specialty = 1)	1.069	-0,176	0.344	0.839	0.583–1.207
Grade (high grade = 1)	1.010	−0.129	0.333	0.879	0.678–1.141
Average monthly household income	1.133	0.076	0.424	1.079	0.895–1.300
Parental education level	1.169	−0.003	0.962	0.997	0.862–1.152
Constant		0.784	0.002	2.190	

**Table 4:** Model 2: multivariate regression analysis of college students’ willingness to participate in community volunteer service for the older adults (*n* = 1,372, −2ll = 1049.17, Nagelkerke *R*^2^ = 0.391).

Variable	VIF	B	P	OR	95%CI
Gender (female = 1)	1.027	0.367	0.024	1.443	1.050–1.984
Origin type (city areas = 1)	1.192	0.082	0.658	1.086	0.755–1.562
Major (medical specialty = 1)	1.115	−0.428	0.061	0.652	0.417–1.019
Grade (high grade = 1)	1.028	0.099	0.538	1.104	0.805–1.514
Average monthly household income	1.137	0.031	0.783	1.032	0.826–1.288
Parental education level	1.184	−0.015	0.869	0.985	0.827–1.174
Capacity building for voluntary older adults care services (good = 1)	1.096	2.528	0.000	12.526	9.153–17.141
School-based support (good = 1)	1.169	0.724	0.000	2.063	1.490–2.856
Social support (good = 1)	1.128	0.336	0.137	1.399	0.899–2.175
The spirit of altruism	3.516	−0.027	0.733	0.974	0.836–1.134
Awareness of respect and care for seniors	3.972	0.260	0.136	1.297	0.921–1.826
Communication abilities	2.603	0.035	0.478	1.036	0.940–1.140
Constant		−2.092	0.000		

## 5 Discussion

Community older adults care is one of the key forms of older adults care in China. As advocates of innovation, college students often introduce fresh perspectives and broader experiences to older adults, earning widespread appreciation for their dedication. A critical challenge in advancing this initiative lies in fostering college students’ commitment to volunteerism—encouraging active participation in community-led services for the older adults, which not only benefits society but also enriches their personal growth. Addressing this challenge will be pivotal to the future development of such programs.

The findings of this study indicate that female college students exhibit greater willingness to engage in community volunteer service for the older adults compared to their male counterparts. When participating in social service programs, male participants prefer roles demanding specialized technical expertise or physical exertion, ranging from paramedical assistance and vocational computer training to community construction and environmental conservation projects; female participants are more receptive to care-based posts centered on empathy and interpersonal communication with older adults, encompassing daily custodial care, emotional support and elder companionship (10, 11). These results align with existing research. For instance, Zhao Xia’s work highlights how innate feminine traits such as emotional sensitivity and compassion motivate women to actively participate in nurturing activities like assisting orphans and the disadvantaged (12–14). Furthermore, studies by Shi et al. (15) reinforce these observations, demonstrating that female college students are more inclined than males to contribute to emergency volunteer initiatives.

Service capacity development significantly influences college students’ willingness to engage in community volunteer services for the older adults. This capacity comprises two key dimensions: (1) mastery of geriatric nursing theories and (2) practical skills and hands-on experience in older adults care. This study revealed that a high proportion of university students were willing to participate in community-based older adults care volunteer services and expressed interest in receiving training in this field. In contrast, only a low proportion of respondents had received promotional information or training related to older adults care services in the past year. This gap highlights a critical deficiency in service-related competencies, which has dampened students’ enthusiasm to varying degrees. Therefore, universities should prioritize enhancing students’ service capabilities in older adults care. First, they should reasonably structure relevant courses, integrating geriatric care-related courses into the public elective or general education systems to ensure systematic mastery of theoretical knowledge in older adults care. Second, regular specialized training programs in older adults care services should be conducted. On one hand, inviting senior caregivers and industry experts from older adults care institutions to deliver lecture on a special subject and skill trainings focused on core competencies such as daily care, health monitoring, and psychological support for the older adults; on the other hand, adopting a hybrid “online + offline” training approach, with offline sessions for hands-on practice and online platforms for live training sessions and Q&A groups, combining service simulations with theoretical courses, and conducting regular mock drills with evaluations. Third, establishing diversified practical platforms for older adults care services is essential. Long-term partnerships should be formed with community older adults care centers and nursing homes to co-build “University Student Older adults Care Service Practice Bases” Regular offline practical activities should be organized, and during internship programs, “simulated older adults care service training” should be offered, utilizing university laboratory resources to create simulated care scenarios, enabling students to practice nursing operations in a simulated environment and enhance their practical skills (16–18). Fourth, establish a comprehensive evaluation mechanism for college students’ volunteer services in community older adults care and improve service capacity and quality continuously based on evaluation feedback. The all-round assessment covers volunteers’ professional proficiency, service attitude and practical capability, the matching degree between provided services and the actual needs of the older adults, the rationality of service procedures, seniors’ satisfaction and sense of gain, as well as comprehensive social impacts. Problems identified from evaluation will be analyzed to formulate targeted improvement strategies, so as to sustainably upgrade volunteers’ service competence and service quality.

School support is the second most significant factor influencing college students’ willingness to participate in community volunteer service for the older adults. Multivariate regression analysis reveals that students who are organized by their colleges to engage in such activities and receive rewards for participation are more likely to take part in community volunteer service for the older adults. This study reveals that the majority of college students prioritize specific forms of recognition for their involvement in community volunteer services for the older adults. Among the desired rewards, 74.05% prefer credit-based incentives, 72.96% seek certificates of honor, 53.79% value corresponding subsidies, and only 3.28% opt for other categories. These findings underscore the need for educational institutions to enhance their support systems for student participation in such initiatives. Universities should strengthen support for college students’ participation in community older adults care volunteer services and enhance collaboration with government departments, communities, and older adults care institutions. First, establishing a coordinated mechanism for volunteer service organizations. A “College Students’ Community Endowment Volunteer Service Coordination Group” should be formed at the school level to coordinate cooperation among departments, student associations, and community older adults care institutions. Regular volunteer service plans should be developed, and volunteer teams should be organized by departments and majors, with guidance teachers responsible for activity planning, safety assurance, and process management. Additionally, a regular communication mechanism between the school and the community should be established to promptly understand older adults care service needs, precisely match students’ volunteer capabilities, and improve the effectiveness of organized participation. Second, optimizing a tiered and categorized reward system. Diversified reward schemes should be designed based on student needs: first, implement credit incentives by incorporating qualified community older adults care volunteer service hours into general education credits or social practice credits, clarifying credit conversion standards and making them public; second, improving honor-based incentives by adding new awards such as “Advanced Collective for Community Older adults Care Volunteer Services” and “Star Volunteers” in addition to existing awards, publicly recognizing outstanding individuals at opening and graduation ceremonies to enhance student pride; third, providing diverse subsidies, including transportation and meal allowances for participants, additional living subsidies for economically disadvantaged outstanding volunteers, and joint efforts with social resources to offer internship recommendations, training coupons, and other value-added rewards for volunteers (19–23).

Additionally, to foster college students’ sustained participation in community-based older adults care volunteer services and avoid superficial or short-term engagement, universities should establish long-term mechanisms to continuously stimulate intrinsic motivation. Specific measures include: First, improving a regularized institutional support system. Integrating older adults care volunteer services into university talent cultivation programs, universities should enforce mandatory requirements combining “volunteer service credits” and practical assessments, stipulate minimum service hours for undergraduate graduation, and incorporate service outcomes into core indicators of comprehensive student evaluations. Standardized procedures for service registration, hour certification, performance evaluation, and commendation should be established, alongside the creation of an archive database to ensure full traceability of student participation. Second, deepening a tripartite collaboration mechanism among universities, community-based older adults care institutions, and enterprises. Universities provide volunteer talent and training resources; older adults care institutions offer service scenarios and demand lists; enterprises donate materials and technical support. The three parties jointly formulate annual service plans, hold regular liaison meetings to address challenges, and jointly establish an “older adults care volunteer development fund” to subsidize students’ transportation and meals, reward outstanding volunteers, and ensure long-term sustainability of the mechanism. Third, strengthening a participatory growth empowerment mechanism. Designing the growth path around students’ self-realization needs, this involves: On one hand, a stepped training mode of “volunteer – core member – leader” is implemented to select outstanding volunteers to participate in the planning and management of service projects, thereby enhancing their leadership skills. On the other hand, the program connects volunteer services with career development by offering internship recommendations in the older adults care industry and discounted vocational certification opportunities to participating students. For those interested in pursuing careers in older adults care, dedicated “order-based classes” are established to foster a virtuous cycle between volunteer service and professional growth. Fourth, establishing a dynamic feedback mechanism. Regularly collecting student feedback on service content, training formats, and incentive measures through online surveys and offline seminars, while implementing a three-party evaluation system involving service recipients, volunteers, and schools. Older adults individuals and their families rate service quality, which is combined with self-assessments by students and peer evaluations by schools to form comprehensive evaluation results. Adjustments to service content and incentive methods are made based on this feedback to ensure alignment with practical needs.

Additionally, government and societal support is critical, including policy and financial backing, increased publicity for volunteer groups, promotion of altruistic and filial social norms, enhancement of volunteers’ recognition and sense of achievement, and legal safeguards for their rights. Through collaborative efforts, these measures aim to stimulate students’ initiative and drive the sustained, long-term development of community-based older adults care volunteer services (24, 25).

We acknowledge key limitations in our research and suggest directions for future studies. This study only recruited undergraduate students from Chinese universities, including medical majors. Due to differences in higher education systems between China and other countries, the research findings have limited cross-national generalizability. Medical students possess more professional knowledge and skills in older adults care, which may cause potential sample bias. In addition, self-reported questionnaire data may introduce self-report bias. Future research can adopt mixed methods such as field observation and third-party evaluation to verify the findings. Moreover, non-response bias should be further explored in follow-up studies by targeted investigation of non-participants and adoption of more diversified sampling methods.

## Data Availability

The raw data supporting the conclusions of this article will be made available by the authors, without undue reservation.
